# Supplementary material for: Single Cell Transcriptomic and Chromatin Profiles Suggest Layer Vb Is the Only Layer With Shared Excitatory Cell Types in the Medial and Lateral Entorhinal Cortex
Source: Front Neural Circuits. 2022 Jan 26;15:806154. doi: 10.3389/fncir.2021.806154 (PMC8826650; doi:10.3389/fncir.2021.806154)
Supplement: Supplementary file 14 [file Data_Sheet_1.ZIP › Sup_vs_Deep_Motifs/homerResults.html]

Sup\_vs\_Deep\_Motifs// - Homer de novo Motif Results


# Homer *de novo* Motif Results (Sup\_vs\_Deep\_Motifs//)

Known Motif Enrichment Results  
Gene Ontology Enrichment Results  
If Homer is having trouble matching a motif to a known motif, try copy/pasting the matrix file into
STAMP  
More information on motif finding results: HOMER
| Description of Results
| Tips
  
Total target sequences = 33511  
Total background sequences = 8540  
\* - possible false positive  

|  |  |  |  |  |  |  |  |  |
| --- | --- | --- | --- | --- | --- | --- | --- | --- |
| Rank | Motif | P-value | log P-pvalue | % of Targets | % of Background | STD(Bg STD) | Best Match/Details | Motif File |
| 1 | G A C T T A C G T G A C T C G A C A T G T A C G C A G T C T A G A G T C C G T A | 1e-3125 | -7.197e+03 | 40.53% | 13.86% | 224.2bp (323.3bp) | E2A(bHLH),near\_PU.1/Bcell-PU.1-ChIP-Seq(GSE21512)/Homer(0.989) More Information \| Similar Motifs Found | motif file (matrix) |
| 2 | A G C T C G A T C T A G A C G T A G C T A C G T C T G A A G C T G T C A G C A T | 1e-326 | -7.508e+02 | 42.52% | 32.42% | 242.3bp (264.0bp) | MF0005.1\_Forkhead\_class/Jaspar(0.951) More Information \| Similar Motifs Found | motif file (matrix) |
| 3 | C A T G T C G A A C G T A C T G C G T A T A C G A G C T G T A C C G T A A G C T | 1e-248 | -5.728e+02 | 13.16% | 7.78% | 234.0bp (268.6bp) | Fra1(bZIP)/BT549-Fra1-ChIP-Seq(GSE46166)/Homer(0.991) More Information \| Similar Motifs Found | motif file (matrix) |
| 4 | C A T G G T A C G C A T T C G A G C T A C G A T A G C T C G T A C G T A C T A G C T A G G C A T | 1e-135 | -3.126e+02 | 8.19% | 4.99% | 230.9bp (262.9bp) | ARGFX/MA1463.1/Jaspar(0.849) More Information \| Similar Motifs Found | motif file (matrix) |
| 5 | A G T C G A T C C G A T C A T G A C T G A G C T C A G T A C T G A G C T C A G T A C T G A G T C | 1e-104 | -2.406e+02 | 0.38% | 0.04% | 261.3bp (202.1bp) | Klf4(Zf)/mES-Klf4-ChIP-Seq(GSE11431)/Homer(0.620) More Information \| Similar Motifs Found | motif file (matrix) |
| 6 | C A T G G A C T C A G T A C T G G A T C G C A T A G T C G C T A C G T A C A G T A C G T G A T C | 1e-90 | -2.085e+02 | 0.28% | 0.02% | 220.5bp (169.8bp) | CEBP(bZIP)/ThioMac-CEBPb-ChIP-Seq(GSE21512)/Homer(0.745) More Information \| Similar Motifs Found | motif file (matrix) |
| 7 | C G T A A G T C C G T A C G T A C G T A C G T A A G T C C G T A C G T A A C G T C G T A A G T C | 1e-80 | -1.848e+02 | 11.93% | 8.84% | 247.1bp (275.4bp) | PB0123.1\_Foxl1\_2/Jaspar(0.749) More Information \| Similar Motifs Found | motif file (matrix) |
| 8 | A C G T A C G T C G T A A C G T A C T G A G T C C G T A C G T A C G T A A C G T | 1e-74 | -1.722e+02 | 1.33% | 0.49% | 238.8bp (191.1bp) | Oct4(POU,Homeobox)/mES-Oct4-ChIP-Seq(GSE11431)/Homer(0.967) More Information \| Similar Motifs Found | motif file (matrix) |
| 9 | C A T G C A G T A T G C G A T C G A T C G C T A A G C T C A G T A C T G G C A T G A C T C G T A | 1e-73 | -1.682e+02 | 0.24% | 0.02% | 232.8bp (26.7bp) | SOX2/MA0143.4/Jaspar(0.811) More Information \| Similar Motifs Found | motif file (matrix) |
| 10 | C G A T A T C G A G T C C G T A A C T G C T A G C G A T G C T A A G T C G T C A C G T A G T A C | 1e-70 | -1.622e+02 | 0.23% | 0.02% | 269.6bp (0.0bp) | AR-halfsite(NR)/LNCaP-AR-ChIP-Seq(GSE27824)/Homer(0.656) More Information \| Similar Motifs Found | motif file (matrix) |
| 11 | T G C A C T G A G T A C G C T A A T G C G T A C G C A T A C G T T G C A C G A T C A T G G A T C | 1e-69 | -1.605e+02 | 0.58% | 0.13% | 243.4bp (302.4bp) | TBX5/MA0807.1/Jaspar(0.668) More Information \| Similar Motifs Found | motif file (matrix) |
| 12 | G A T C T A C G G T A C C G T A C G T A C G A T A G C T C T G A | 1e-65 | -1.506e+02 | 8.26% | 5.93% | 244.0bp (258.0bp) | DLX5/MA1476.1/Jaspar(0.942) More Information \| Similar Motifs Found | motif file (matrix) |
| 13 | T C G A T A G C G T C A C G T A T C A G C G T A C T A G C T A G C T A G T C A G T G A C C G T A | 1e-57 | -1.329e+02 | 0.20% | 0.02% | 215.6bp (77.1bp) | SOX15/MA1152.1/Jaspar(0.603) More Information \| Similar Motifs Found | motif file (matrix) |
| 14 | A G T C A G C T A C T G A C G T C G T A G T C A C G T A A G T C A G T C A C G T | 1e-53 | -1.222e+02 | 0.53% | 0.14% | 229.3bp (306.4bp) | FOXA1/MA0148.4/Jaspar(0.716) More Information \| Similar Motifs Found | motif file (matrix) |
| 15 | A C G T A G T C A C T G A G T C A C T G G C T A A C T G A C G T | 1e-48 | -1.124e+02 | 0.51% | 0.14% | 251.6bp (249.2bp) | ZBTB33/MA0527.1/Jaspar(0.752) More Information \| Similar Motifs Found | motif file (matrix) |
| 16 | C A T G A T G C G C T A T C A G G C A T A C T G G A C T T G C A G A T C G C A T A T G C G T A C | 1e-47 | -1.100e+02 | 0.27% | 0.04% | 215.6bp (250.1bp) | PB0091.1\_Zbtb3\_1/Jaspar(0.693) More Information \| Similar Motifs Found | motif file (matrix) |
| 17 | A T G C A C T G A C T G G A C T A T G C T C G A G A C T C A G T | 1e-42 | -9.782e+01 | 4.11% | 2.79% | 264.5bp (319.8bp) | NR1H4/MA1110.1/Jaspar(0.927) More Information \| Similar Motifs Found | motif file (matrix) |
| 18 | C T A G A G C T C A T G C G T A A G T C A C T G A G C T G A T C | 1e-40 | -9.316e+01 | 2.27% | 1.36% | 245.3bp (250.5bp) | CREB3L4(var.2)/MA1475.1/Jaspar(0.855) More Information \| Similar Motifs Found | motif file (matrix) |
| 19 | A C T G C T G A A G C T A T G C A G T C A T C G A C T G G T A C | 1e-38 | -8.930e+01 | 0.39% | 0.09% | 286.1bp (338.3bp) | PB0153.1\_Nr2f2\_2/Jaspar(0.713) More Information \| Similar Motifs Found | motif file (matrix) |
| 20 | A G T C C G T A A G T C A G T C A G T C A G T C C G T A A C G T C G T A A C T G A C G T A G T C | 1e-37 | -8.615e+01 | 0.24% | 0.04% | 227.5bp (176.0bp) | SREBF2/MA0596.1/Jaspar(0.688) More Information \| Similar Motifs Found | motif file (matrix) |
| 21 | C G T A A C G T C G T A A G T C A G T C A C G T A C T G A C T G A G T C A C G T | 1e-28 | -6.590e+01 | 0.20% | 0.04% | 238.0bp (482.0bp) | ZEB1(Zf)/PDAC-ZEB1-ChIP-Seq(GSE64557)/Homer(0.742) More Information \| Similar Motifs Found | motif file (matrix) |
| 22 | A G T C A G T C A G T C C G T A A C T G A C G T C G T A C G T A A C T G C G T A A G T C A G T C | 1e-26 | -6.216e+01 | 0.25% | 0.06% | 237.6bp (308.7bp) | HOXD13/MA0909.2/Jaspar(0.617) More Information \| Similar Motifs Found | motif file (matrix) |
| 23 | A C T G T G A C A G T C A C T G A C T G G A T C G T A C A T C G | 1e-26 | -6.108e+01 | 1.56% | 0.94% | 269.4bp (345.5bp) | YY2/MA0748.2/Jaspar(0.616) More Information \| Similar Motifs Found | motif file (matrix) |
| 24 | A G T C A G T C A G T C A C G T C G T A C G T A A G T C A G T C A G T C A C G T C G T A A C G T | 1e-21 | -4.901e+01 | 0.17% | 0.04% | 244.8bp (244.6bp) | Crx/MA0467.1/Jaspar(0.625) More Information \| Similar Motifs Found | motif file (matrix) |
| 25 \* | A G T C A C G T A C T G A G T C A C G T A C T G C G T A A C T G A C T G A C G T | 1e-11 | -2.607e+01 | 0.23% | 0.10% | 274.7bp (243.5bp) | Unknown-ESC-element(?)/mES-Nanog-ChIP-Seq(GSE11724)/Homer(0.740) More Information \| Similar Motifs Found | motif file (matrix) |
| 26 \* | A C G T A G T C A C G T A C T G A C T G A C T G A C T G A G T C A G T C A C G T | 1e-10 | -2.364e+01 | 0.20% | 0.09% | 254.7bp (393.0bp) | Plagl1/MA1615.1/Jaspar(0.765) More Information \| Similar Motifs Found | motif file (matrix) |
| 27 \* | A G T C A G C T A G T C C G A T A G T C G A C T A G T C A G C T A T G C A G C T A T G C A G C T | 1e-7 | -1.658e+01 | 4.03% | 3.50% | 259.5bp (291.7bp) | PRDM1/MA0508.3/Jaspar(0.686) More Information \| Similar Motifs Found | motif file (matrix) |
| 28 \* | C G T A A C G T A C G T A C G T C G T A C G T A A C G T A C T G C G T A C G T A | 1e-4 | -1.070e+01 | 0.39% | 0.28% | 230.9bp (288.5bp) | PB0005.1\_Bbx\_1/Jaspar(0.775) More Information \| Similar Motifs Found | motif file (matrix) |
